# Neural inhibition for continual learning and memory

**DOI:** 10.1016/j.conb.2020.09.007

**Published:** 2021-04

**Authors:** Helen C Barron

**Affiliations:** 1 Medical Research Council Brain Network Dynamics Unit, Nuffield Department of Clinical Neurosciences, University of Oxford, Mansfield Road, Oxford, OX1 3TH, UK; 2 Wellcome Centre for Integrative Neuroimaging, University of Oxford, FMRIB, John Radcliffe Hospital, Oxford, OX3 9DU, UK

## Abstract

Humans are able to continually learn new information and acquire skills that meet the demands of an ever-changing environment. Yet, this new learning does not necessarily occur at the expense of old memories. The specialised biological mechanisms that permit continual learning in humans and other mammals are not fully understood. Here I explore the possibility that neural inhibition plays an important role. I present recent findings from studies in humans that suggest inhibition regulates the stability of neural networks to gate cortical plasticity and memory retrieval. These studies use non-invasive methods to obtain an indirect measure of neural inhibition and corroborate comparable findings in animals. Together these studies reveal a model whereby neural inhibition protects memories from interference to permit continual learning. Neural inhibition may, therefore, play a critical role in the computations that underlie higher-order cognition and adaptive behaviour.

## Highlights

• In humans, the concentration of cortical GABA decreases during learning.
• Inhibitory plasticity provides homeostatic control to restore network stability.
• Memories are held dormant unless latent inhibitory connections are unmasked.
• Cortical inhibition protects overlapping memories from interference.
• The emerging model suggests neural inhibition promotes continual learning.

**Current Opinion in Neurobiology** 2021, **67**:85–94

This review comes from a themed issue on **Neurobiology of learning and plasticity**

Edited by **Tara Keck** and **Sheena A Josselyn**

For a complete overview see the Issue and the Editorial

Available online 28th October 2020

**https://doi.org/10.1016/j.conb.2020.09.007**

0959-4388/© 2020 The Author(s). Published by Elsevier Ltd. This is an open access article under the CC BY license (http://creativecommons.org/licenses/by/4.0/).

## Introduction

The term inhibition is embedded in our language and in our thinking. But what does it mean? In psychology, inhibition is often used to refer to a process that involves withholding or preventing a thought, memory or action from being expressed. In physiology, inhibition refers to a process whereby neural activity patterns are suppressed, blocked or restricted in both space and time. In the mammalian brain, inhibition is predominantly realised by inhibitory interneurons which release gamma amino butyric acid (GABA), the principle inhibitory neurotransmitter.

**Figure 1:**
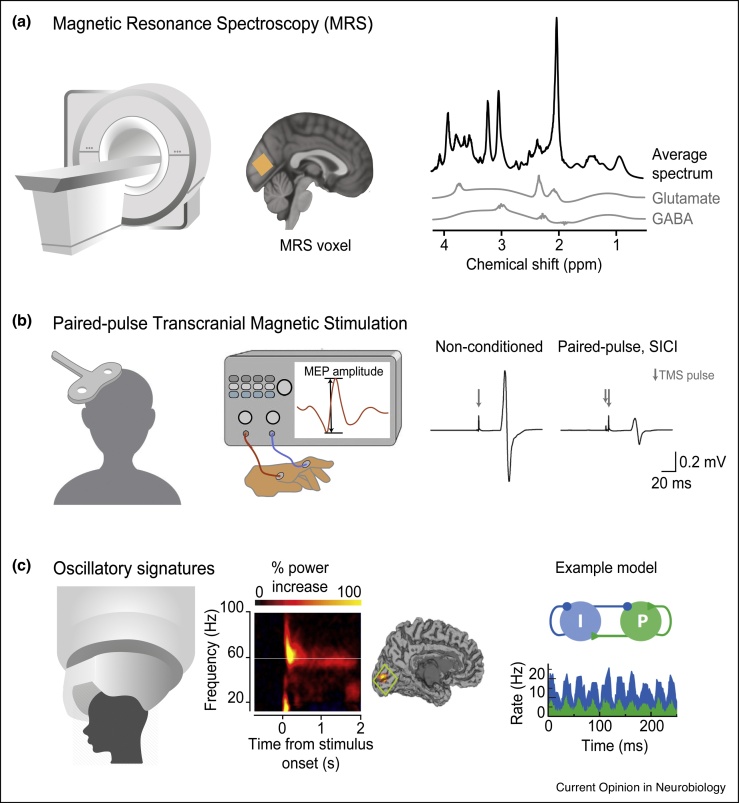
Non-invasive measures of neural inhibition. **(a)**^1^H Magnetic Resonance Spectroscopy (MRS): MRS is acquired within an MRI scanner (left panel), from a region of interest here illustrated by a 2 × 2  × 2 cm^2^ voxel in primary visual cortex (middle panel, voxel in orange). With suppression of the water peak, the concentration of different metabolites can be estimated from the acquired spectra, including the concentration of glutamate and GABA (right panel). ‘ppm’ indicates parts-per-million. See Box 1 for more detail. **(b)** Paired-pulse transcranial magnetic stimulation (TMS): TMS involves using a rapidly changing magnetic field to induce electrical current in the underlying neural tissue (left panel). When TMS is applied to motor cortex, an electromyography trace can be recorded from the contralateral first dorsal interosseus muscle in the hand, to quantify the motor-evoked potential (MEP) induced by a TMS pulse (middle panel). When a conditioning pulse precedes a test pulse by ∼3 ms the resulting MEP amplitude is markedly reduced, a protocol described as short-interval intracortical inhibition (SICI). See Box 1 for more detail. **(c)** Oscillatory signatures: Neural oscillations can be recorded using magnetoencephalography (MEG) (left panel) or electroencephalography (EEG). For example, in response to a simple visual grating stimulus, time-frequency reconstruction of MEG data reveals an increase in gamma power localised to visual cortex (middle panel), modified from Ref. [61]. Gamma oscillations can be modelled using a simple reciprocally connected excitatory (P) and inhibitory (I) pair of neurons, with fast excitation and delayed feedback inhibition (right panel), modified from Ref. [62].

Direct measures of neural inhibition *in vivo* are restricted to invasive recordings which, except in rare circumstances, can only be performed in animal models. By contrast, most non-invasive tools for measuring human brain activity, including functional Magnetic Resonance Imaging (fMRI), aggregate the response from excitatory and inhibitory signals across large neural populations [1]. However, to establish how inhibitory processing contributes to higher-level cognition and neuropsychiatric disease, processes that cannot easily be investigated in animal models, there is growing demand for non-invasive measures of neural inhibition. As outlined in Figure 1 and Box 1, several non-invasive tools are now being used to obtain indirect measures of neural inhibition in the human brain, including: (1) ^1^H magnetic resonance spectroscopy (^1^H MRS); (2) brain stimulation; and (3) oscillatory signatures.

Box 1 **Non-invasive tools for measuring neural inhibition in humans**

**^1^H MRS**: ^1^H MRS provides a non-invasive means to detect and quantify chemical compounds within living tissue. In humans, MRS can be used to quantify the concentration of GABA within discrete regions of the brain, together with other neurochemicals such as glutamate (Figure 1a). The concentration of GABA in human brain tissue is relatively low, and the spectral peaks for GABA overlap with other, more abundant neurochemicals. However ultra-high field MRI (7T and above) increases spectral resolution, allowing for more reliable detection [63].

Interpreting MRS measures of GABA is not straightforward. Only a fraction of MRS-derived neurometabolite concentration reflects neurotransmitter release as the release and recycling of glutamate and GABA constitute major metabolic pathways. Moreover, the temporal resolution of MRS is insufficient to account for changes in GABA that occur on a timescale described by synaptic activity. Meaningful interpretation of MRS-derived glutamate and GABA are nevertheless supported by a major body of work showing an approximately 1:1 relationship between the rate of glutamine-glutamate cycling, which is necessary for glutamate and GABA synthesis, and neuronal oxidative glucose consumption, which indirectly supports neurotransmitter release among other processes [64,65]. Changes in the concentration of MRS-derived GABA may therefore reflect, if indirectly, physiological changes in inhibition at the microcircuit-level.

**Brain stimulation:** Paired-pulse transcranial magnetic stimulation (ppTMS) can provide a non-invasive readout for GABAergic inhibition in the human brain. Inhibitory ppTMS involves delivering a conditioning stimulus shortly before a test stimulus, with the two pulses separated by either 2−4 ms (short-intracortical inhibition, SICI) or 100−200 ms (long-intracortical cortical inhibition, LICI). When applied to primary motor cortex (M1), the test stimulus in an inhibitory ppTMS protocol elicits a suppressed motor evoked potential (MEP) in the target muscle, relative to an unconditioned test stimulus (Figure 1b). Pharmacological manipulations suggest the inter-pulse interval determines whether the readout predominantly reflects GABA-A or GABA-B receptor mediated inhibition, with SICI mediated by GABA-A receptors [66], and LICI mediated by GABA-B receptors [67]. While this approach has high temporal resolution, it is largely restricted to measuring neural inhibition in M1.

**Oscillatory signatures:** Another putative index for neural inhibition is provided by oscillations, which can be measured noninvasively at the scalp using EEG or MEG (Figure 1c). Studies in both animals and humans suggest oscillations in the gamma-frequency band (30−90 Hz) reflect underlying GABAergic activity. Specifically, gamma oscillations appear to be mediated by synchronous inhibitory post-synaptic potentials in excitatory pyramidal cells, brought about by fast-spiking parvalbumin positive (PV+) inhibitory interneurons [62,68]. In the human brain, the resting GABA concentration measured from primary visual cortex using MRS can predict the peak frequency of gamma oscillations in response to visual stimulation [61].

Alt-text: Box 1

Unlike invasive tools available in animal models, these non-invasive methods typically have poor spatiotemporal resolution and cannot dissociate different inhibitory interneuron subtypes, which show great diversity in their connectivity profiles and cellular properties [2,3]. It is, therefore, necessary to exercise caution when interpreting data acquired using non-invasive measures. Nevertheless, the studies illustrated below describe a clear mechanistic role for neural inhibition in human learning and memory. Specifically, in the cortex, inhibition gates learning before stabilising and protecting newly formed memories from interference. This phenomenon is observed across a number of different cortical regions, pointing to a canonical microcircuit mechanism which may involve a disinhibitory circuit motif. Neural inhibition, therefore, underpins the central features of human learning and memory, namely our ability to continually learn across our lifespan while retaining the capacity to recall hundreds of thousands of different memories.

## Gating learning: decreasing inhibition

Studies performed in animal models demonstrate that a reduction in GABAergic tone is necessary for induction of neocortical plasticity via long-term potentiation (LTP) [4,5]. Neural inhibition may therefore, gate plasticity that accompanies new learning, by allowing cortical maps to be reshaped when latent inhibition is unmasked [6].

**Figure 2:**
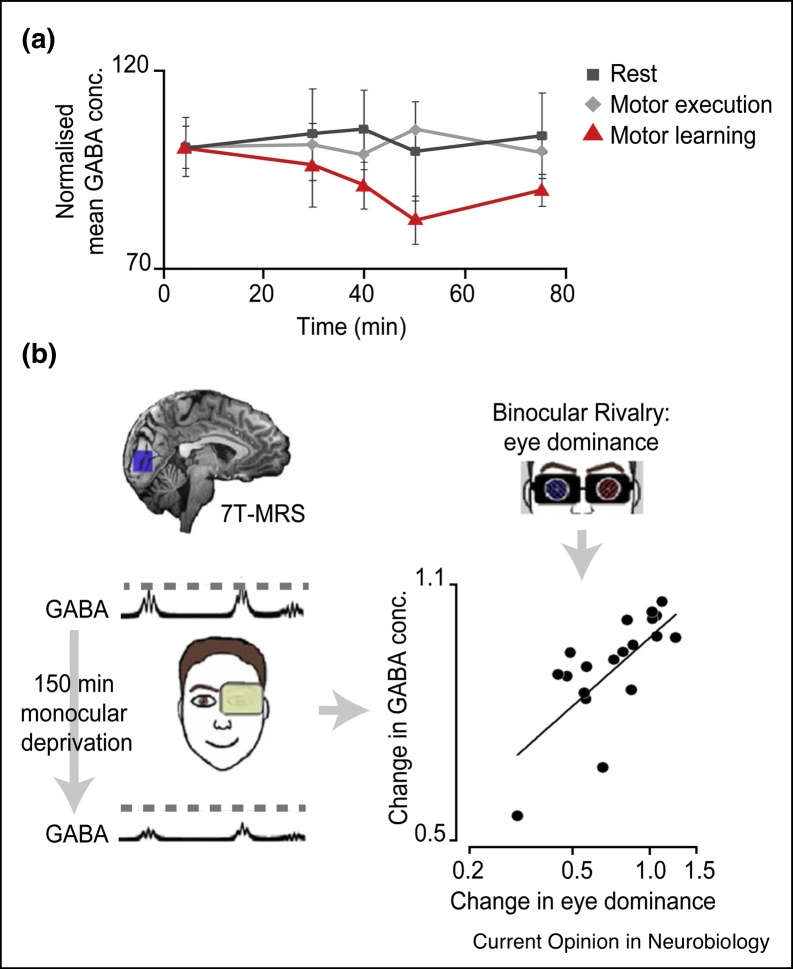
Neural inhibition decreases during learning. **(a)** During motor learning of a sequence tracking task (red), a decrease in the concentration of MRS-measured GABA is observed in sensorimotor cortex. This decrease in GABA is not observed during a motor task that does not require learning (light grey), nor during rest (dark grey). Modified from Ref. [7^••^]. **(b)** Following 2.5 hours of monocular deprivation, a significant reduction in the concentration of GABA is observed in primary visual cortex, measured using MRS. The relative change in GABA predicts the resulting change in eye dominance measured using a binocular rivalry paradigm. Modified from Ref. [15^•^]. ‘conc.’ refers to ‘concentration’.

In the human brain, a non-invasive signature of this process can be acquired using MRS (Figure 1a, Box 1). For example, during motor learning a rapid, reversible decrease in MRS-derived GABA concentration can be observed in sensorimotor cortex [7^••^] (Figure 2a). This decrease in GABA is selective to the contralateral hemisphere of the moved hand and is not observed during control tasks that require equivalent force generation but lack a learning component. Similar reductions in inhibition have been observed when MRS is used to track changes in GABA in primary motor cortex that accompany motor skill acquisition [8,9], or when using paired-pulse transcranial magnetic stimulation (ppTMS) to probe intracortical inhibition during early phases of learning [10] (Figure 1b, Box 1).

Further evidence to suggest a decrease in local inhibition is necessary for new learning derives from causal approaches. For example, pharmacological studies (Box 2) show evidence for reduced motor skill acquisition when human participants are premedicated with the GABA agonist lorazepam [11]. Conversely, motor learning is improved when anodal transcranial direct-current stimulation (tDCS) is applied to primary motor cortex during learning [12,13], a tool known to induce a reduction in the concentration of cortical GABA (Box 2). Inter-individual differences support this view: following application of anodal tDCS to primary motor cortex, subjects that exhibit a large reduction in MRS-derived GABA perform better on a motor learning task using robotic force adaptation [14^•^].

Box 2 **Tools for manipulating neural inhibition in humans**

**Brain stimulation:** Trans-cranial direct current stimulation (tDCS) is a non-invasive method used to modulate the concentration of cortical GABA [69]. During and following tDCS, cortical excitability is enhanced. This can be measured by local neuronal firing rates [70] or using non-invasive ppTMS [71] (Figure 1b, Box 1). This increase in cortical excitability is sustained after stimulation for minutes to hours [70] via a protein synthesis dependent process [72]. MRS measured during or after tDCS suggests this increase in excitability can be attributed to a decrease in the concentration of cortical GABA [14^•^,39,46^••^,73^•^].

**Pharmacological approaches:** Pharmacological approaches can be used to modulate neural inhibition in humans. This typically involves using a randomised double-blind placebo-controlled experimental design. GABA agonists, which include drugs such as benzodiazepines and baclofen, enhance GABA_A_ and GABA_B_ receptor mediated neurotransmission, respectively. GABA antagonists, which decrease the effect of GABAergic neurotransmission, include drugs such as flumazenil. However, due to the stimulant and convulsant effects of GABA antagonists, the use of GABA antagonists is typically restricted to clinical settings to counteract overdose of sedative drugs.

Alt-text: Box 2

Most studies investigating the role of neural inhibition in human learning have focused on multi-trial learning in motor cortex, where a reduction in inhibition may promote integration across multiple discrete experiences. These findings may reflect a more general mechanism as plasticity in other brain regions reveals a similar profile. For example, following 2.5 hours of monocular deprivation, a protocol used to induce homeostatic plasticity, MRS reveals a significant reduction in MRS-derived GABA in primary visual cortex. Moreover, the relative change in GABA reliably predicts the resulting change in eye dominance [15^•^] (Figure 2b).

A key limitation of non-invasive methods is that they cannot identify the contribution made by different interneurons subtypes. Instead we must rely on genetic techniques available in animal models which suggest new learning is gated by several different inhibitory interneuron subtypes [16,17]. In auditory and prefrontal cortex, for example, the unconditioned stimulus in auditory fear conditioning rapidly recruits vasoactive intestinal peptide (VIP+) interneurons, which in turn target parvalbumin (PV+) and somatostatin-positive (SOM+) inhibitory interneurons to release excitatory pyramidal cells from inhibition [18,19]. This disinhibitory circuit motif may be ubiquitous in cortex, supporting fear conditioning, which shares many features with episodic memory, but also multi-trial learning [20, 21, 22, 23]. This raises the intriguing possibility that disinhibitory circuit motifs underlie the reduction in GABA reported in humans during multi-trial motor learning.

While reduced neural inhibition by disinhibitory circuits motifs may provide a general mechanism for new learning, there are some notable exceptions. For example, in human primary visual cortex (V1), learning dependent changes in MRS-derived GABA reveal disinhibition during a target-detection task but increased inhibition during feature discrimination [24]. Another exception is reported in the dCA1 region of the hippocampus in mice, where the unconditioned stimulus in a fear conditioning paradigm activates SOM+ interneurons to suppress activity in pyramidal cells [25]. Similarly, in the human hippocampus, invasive recordings in epilepsy patients suggest successful encoding of verbal memory is predicted by an increase in gamma oscillations [26], a putative index for neural inhibition (Figure 1c, Box 1). One possibility is that these inhibitory signatures in the hippocampus reflect a distinct mechanism, where an increase in neural inhibition is necessary to pattern separate and resolve mnemonic information for rapid encoding of episodic memory. Neural network modelling supports this view, showing that inhibitory interneurons play a key role in controlling features of hippocampal activity deemed necessary for one-shot learning [27]. Therefore, while new learning is typically accompanied by reduced neural inhibition, this phenomenon is sensitive to the precise brain region and task demands.

## Restoring stability: inhibitory plasticity

Despite fluctuations in neural inhibition with new learning, empirical work in animal models [28,29] together with theoretical modelling [30,31] reveals a tight balance between excitation and inhibition (EI) in cortical circuits. Thus, on average, the excitatory input received by a neuron is cancelled out by equivalent inhibitory input. This ensures neurons and networks are neither hypo-excitable nor hyper-excitable for prolonged periods. After new learning, when a decrease in neural inhibition introduces network instability, homeostatic mechanisms must be employed to restore net activity around a set point.

Theoretical models suggest various plasticity mechanisms work in concert to provide homeostatic control [32]. Broadly, these homeostatic mechanisms may apply to either excitatory synapses, via heterosynaptic plasticity rules where the strength of excitatory synapses are scaled up or down [33], or to inhibitory synapses, via inhibitory plasticity which has emerged as a major mechanism to stabilise neural networks [34]. Rather than merely promoting global stability, recordings in rodents suggest inhibitory plasticity promotes stimulus-specific changes in inhibition. For example, inhibitory currents can become tuned to a particular stimulus [35], while subtypes of inhibitory interneuron, specifically parvalbumin positive (PV+) interneurons, may stabilise neural networks to mediate memory consolidation [36]. Theoretical models further demonstrate that when a large number of memories are embedded within a network, selective inhibition is necessary for stability [37]. Inhibitory plasticity may, therefore, modulate inhibitory synapses to selectively match excitatory synaptic changes that accompany new learning.

**Figure 3:**
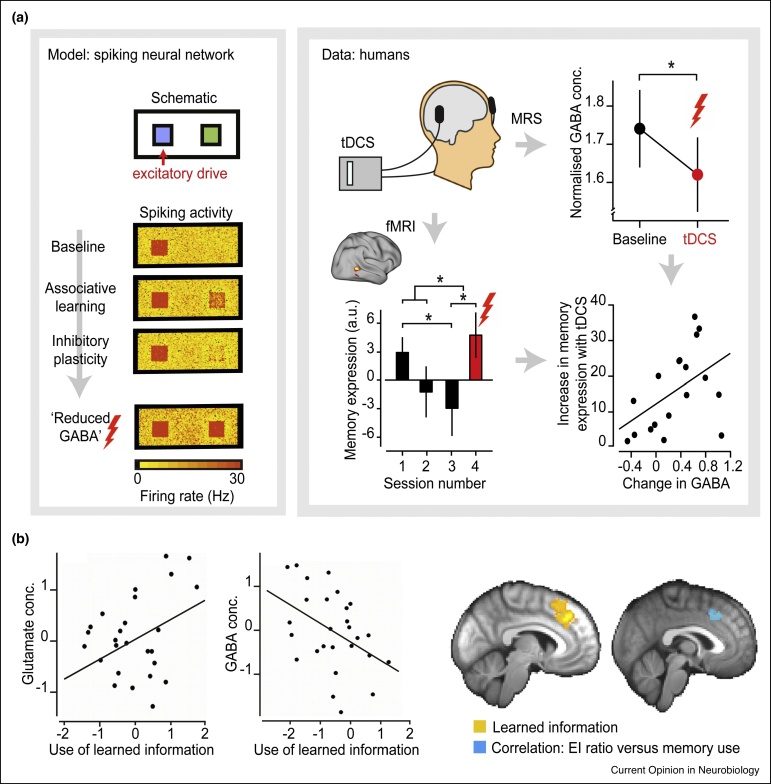
Neural inhibition masks latent memories and mediates retrieval. **(a)** Left panel: A recurrent neural network used to predict cortical memory expression following new learning. Two cell assemblies are embedded in the network (blue and green, see schematic). The spiking activity of the network is probed at four time-points by driving the blue cell assembly (red arrow) and the firing rate of each neuron projected onto a grid. Initially only the blue assembly is activated (‘*Baseline*’), but coactivation can be observed immediately after associative learning (‘*Associative learning*’), where excitatory connections between the blue and green assemblies are strengthened. This coactivation is only temporary as inhibitory plasticity balances surplus excitation, quenching coactivation between the two assemblies (‘*Inhibitory plasticity*’). Coactivation can be recovered when the efficacy of inhibitory synapses are reduced to mimic a reduction in cortical GABA (‘*Reduced GABA*’). Right panel: In humans, predictions from the model are tested. Cortical memory expression measured using representational fMRI [38] is significant immediately after learning (session 1), but becomes silent with time (session 2–3). When anodal tDCS is used to reduce cortical GABA, memories are re-expressed, with the strength of expression predicted by the change in GABA. Modified from Ref. [39^••^]. **(b)** Left panels: The concentration of MRS-measured glutamate and GABA in ACC predicts participants’ use of learned information, a parameter that derives from a behavioural model. Right panels: ACC represents learned information (yellow). Individual differences in the ratio of glutamate to GABA (EI ratio) predict the use of learned information in the same brain region (blue). Modified from Ref. [44^••^] ‘conc.’ refers to ‘concentration’.

By combining non-invasive imaging techniques with brain stimulation and computation modelling, it is now possible to assess the consequences of selective inhibitory synaptic potentiation in the human brain. After participants learn to associate pairs of abstract visual stimuli, the neural expression of these learned associations can be probed using fMRI repetition suppression, a tool used to index neural representations [38]. Immediately after learning, associative memories are expressed in human cortex, but become silent over time, despite persistence in behaviour. When MRS-derived cortical GABA is transiently reduced using anodal tDCS, associative memories are re-expressed in proportion to the change in GABA, over and above a global change in cortical excitability [39^••^] (Figure 3a). These observations are consistent with homeostatic rebalancing via inhibitory plasticity, as illustrated in a spiking network model [40] (Figure 3a).

These results suggest that following learning, inhibitory plasticity restores network stability by forming inhibitory replicas of newly modified excitatory synapses. Memories in cortex may, therefore, be stored in a dormant state, and only expressed when latent inhibitory connections are unmasked. Spiking activity recorded in the songbird corroborates this view: in juvenile birds, the ability to learn a tutor song predicts maturation of synaptic inhibition, while in adult birds premotor neurons remain silent but can be released from inhibitory control via application of the GABA antagonist gabazine [41]. Together, these findings in humans and animals give rise to the concept of an ‘inhibitory engram’ [42] which, unlike ‘silent’ engrams [43], is a population of inhibitory interneurons that undergo plasticity with new learning and subsequently gate memory expression.

## Modulating neural inhibition for memory retrieval

Inhibitory plasticity may, therefore, be considered a key homeostatic mechanism that facilitates stable memory storage. Yet, this model implies that memory retrieval or reinstatement occurs when neural inhibition is reduced, either locally or at a network-wide level. Indeed, theoretical models suggest retrieval may be achieved by perturbing local changes in synaptic input, effectively gating the expression of neural activity via a transient break in EI balance [31].

In humans, it remains difficult to measure dynamic changes in EI balance. However, resting levels of GABA and glutamate may be related to cognitive variables, such as memory retrieval. A particularly effective approach involves using behavioural modelling. For example, during a decision-making task, choices made by each participant can be explained by several parameters in a computational reinforcement learning model. Together with MRS data recorded from the anterior cingulate cortex (ACC) at rest, this reveals a positive relationship between the model parameter that describes ‘use of learned information’ and glutamate in ACC, with an equivalent negative relationships for GABA [44^••^] (Figure 3b). This result suggests that the extent to which memory is used to guide decision-making may in part be determined by resting levels of MRS-derived GABA and glutamate.

Invasive methods available in rodents now provide sufficient spatiotemporal resolution to track dynamic changes in inhibition during memory retrieval. Studies employing these methods suggest latent memories are released from inhibitory control during memory retrieval using the same disinhibitory circuit motif that supports learning [16]. Therefore, in addition to gating new learning, neural inhibition may control the transient release of memory during retrieval. Future research in humans using functional or ‘time-resolved’ MRS [45] may provide a non-invasive signature for disinhibitory circuit mechanims, by tracking dynamic changes in glutamate/GABA ratio.

## Continual learning: protecting memories from interference

The functional significance of incorporating selective neural inhibition in cortical memories may be fully realised when considering how humans and other mammals continue to learn new information throughout their lifetime. While experiences often overlap with each other in content or sensory information, we can selectively recall memories for different experiences. This ability to continually learn new information while protecting memories from interference may in part be supported by the inhibitory component of a memory.

**Figure 4:**
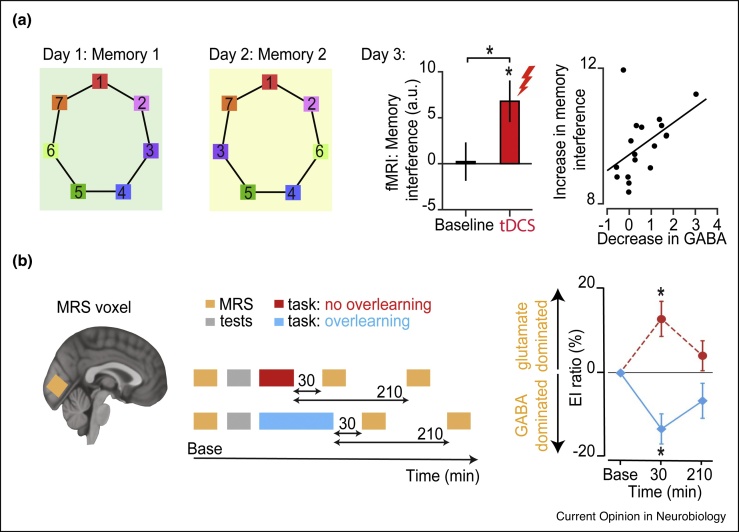
Neural inhibition protects against memory interference. **(a)** Across two consecutive days, participants learned two overlapping memories, memory 1 and memory 2, each of which constituted a set of seven associations. Interference between the two overlapping memories increased when cortical GABA was reduced using anodal tDCS, as measured using representational fMRI [38]. Individual differences in the decrease in GABA predicted participants’ increase in memory interference. Modified from Ref. [46^••^]. **(b)** The concentration of glutamate and GABA were measured at three time points during a visual task, using MRS in primary visual cortex (orange): before learning (‘*Base*’), and 30 min. and 210 min. after learning (‘*30*’ and ‘*210*’, respectively). Extended training on the visual task (‘*overlearning*’, shown in blue) shifted the neurochemical profile of visual cortex from glutamate-dominated excitation (‘*no overlearning*’, red) to GABA-dominated inhibition (‘*overlearning*’, blue). Modified from Ref. [48^••^].

To test this hypothesis in the human brain, Koolschijn *et al.* trained human participants to encode two overlapping but context-dependent memories. Training was performed across two consecutive days [46^••^]. On the third day, ultra-high field 7T MRI was used to measure interference between the two memories, both before and after application of tDCS. When MRS-derived GABA was reduced in neocortex using anodal tDCS, neural memory interference increased (Figure 4a). Moreover, the drop in MRS-derived GABA predicted the increase in memory interference (Figure 4a), which in turn predicted behavioural measures of contextual memory interference. These findings are consistent with observations in rodents where activity in cortical interneurons gates context-dependent behavioural performance [47].

Neural inhibition may therefore play a key role in promoting continual learning by protecting memories from interference. This view is further supported by evidence in humans showing that extensive training, or ‘overlearning’, on a visual task prevents memory interference and shifts the neurochemical profile in visual cortex from glutamate-dominated excitation to GABA-dominated inhibition [48^••^] (Figure 4b).

While inhibitory rebalancing following learning promotes network stability, the ability to generalise may be predicted by the relative instability of memory. Behavioural studies in humans show that generalisation from a motor to a word list task is prevented if learning on the two tasks is separated by a sufficiently long time interval [49]. Intriguingly, this time interval is concomitant with the time course of homeostatic inhibitory rebalancing observed in rats [35]. Generalisation of learned information may, therefore, predominantly occur before inhibitory rebalancing, in the time window where memories are unstable. This gives rise to the possibility that memory stability, as determined by neural inhibition, sets a trade-off between memory interference and our ability to generalise knowledge across different experiences.

Although continual learning can be observed in humans and some animals, this contrasts with artificial neural networks which typically struggle to avoid overfitting and show catastrophic forgetting when trained on multiple tasks. Artificial neural networks may therefore be used as a testing ground to identify those biological constraints necessary for continual learning. This approach reveals the importance of several key biological features, including synaptic complexity [50], ‘experience replay’ [51], and factorised or contextual representations [52, 53, 54]. Incorporating separate excitatory and inhibitory populations [55] and the full diversity of different inhibitory subtypes in artificial neural networks may yield further insight into the functional role of neural inhibition in continual learning.

## Controlling memory: the hippocampus and prefrontal cortex

Current available methods for indexing neural inhibition in the human brain (MRS and TMS) are typically deployed within a single brain region. This makes it difficult to assess how interactions between different brain regions might affect the contribution of neural inhibition to learning and memory. One possibility is that brain regions that reside higher in the cortical hierarchy regulate neural inhibition at lower levels [56]. For example, the hippocampus, which resides at the apex of a sensory processing hierarchy, pattern separates overlapping memories according to contextual cues [46^••^], precisely the signal necessary to control neocortical inhibition during memory retrieval [54]. Indeed, activity in the hippocampus predicts behavioural measures of memory interference, but not if neocortical GABA is manipulated using anodal tDCS [46^••^].

Cognitive tasks that investigate suppression of unwanted thoughts (e.g. ‘think/no think’ paradigms) further support a framework in which the hippocampus together with the prefrontal cortex mediates inhibitory control over memory retrieval. For example, higher resting levels of MRS-derived GABA in the hippocampus predict superior suppression of unwanted thoughts [57^••^]. Moreover, functional imaging studies suggest this suppression of unwanted thoughts involves modulation of hippocampal activity by the lateral prefrontal cortex [58]. These findings may explain why hippocampal hyperactivity, attributed to a deficit in GABAergic inhibition local to the hippocampus, is reported in conditions such as post-traumatic stress disorder, anxiety and schizophrenia, where unwanted or intrusive memories are a core feature [59,60].

## Conclusion

Non-invasive measures of neural inhibition in the human brain suggest inhibitory interneurons gate learning and memory. During multi-trial learning, measures of cortical inhibition acquired using MRS reveal a rapid, reversible decrease in neocortical GABA, where a larger drop in GABA predicts superior learning. Network stability is then restored via homeostatic mechanisms that likely include inhibitory plasticity. This promotes stimulus selective inhibition which holds memories dormant unless latent inhibitory connections are unmasked. Resting levels of GABA measured using MRS can predict how memories are used to guide decision-making, while a decrease in GABA, induced using anodal tDCS, leads to an increase in memory interference. Together these findings describe a model where transient decreases in neural inhibition gate new learning, memory retrieval and even generalisation, while inhibitory rebalancing mitigates against catastrophic forgetting. Information in neural circuits is, therefore, not simply remembered or forgotten but rather subject to an inhibitory brake that provides the mechanistic flexibility necessary for continual learning. This suggests inhibitory processing plays a key role in the neural computations responsible for adaptive cognition and behaviour.

## Conflict of interest statement

Nothing declared.

## References and recommended reading

Papers of particular interest, published within the period of review, have been highlighted as

• of special interest
•• of outstanding interest
